# A Flexible 5-In-1 Microsensor for Internal Microscopic Diagnosis of Vanadium Redox Flow Battery Charging Process

**DOI:** 10.3390/s19051030

**Published:** 2019-02-28

**Authors:** Chi-Yuan Lee, Chin-Lung Hsieh, Chia-Hung Chen, Yen-Pu Huang, Chong-An Jiang, Pei-Chi Wu

**Affiliations:** 1 Department of Mechanical Engineering, Yuan Ze Fuel Cell Center, Yuan Ze University, Taoyuan 320, Taiwan; a45645645630@gmail.com (Y.-P.H.); s102080202@gmail.com (C.-A.J.); s1040802@mail.yzu.edu.tw (P.-C.W.); 2 Institute of Nuclear Energy Research, Taoyuan 325, Taiwan; clhsieh@iner.gov.tw; 3 HOMYTECH Global CO., LTD, Taoyuan 334, Taiwan; sv3@homytech.com

**Keywords:** vanadium redox flow battery, internal real-time microscopic diagnosis, MEMS, flexible five-in-one microsensor

## Abstract

Multiple important physical parameters in the vanadium redox flow battery are difficult to measure accurately, and the multiple important physical parameters (e.g., temperature, flow, voltage, current, pressure, and electrolyte concentration) are correlated with each other; all of them have a critical influence on the performance and life of vanadium redox flow battery. In terms of the feed of fuel to vanadium redox flow battery, the pump conveys electrolytes from the outside to inside for reaction. As the performance of vanadium redox flow battery can be tested only by an external machine—after which, the speed of pump is adjusted to control the flow velocity of electrolyte—the optimum performance cannot be obtained. There is a demand for internal real-time microscopic diagnosis of vanadium redox flow batteries, and this study uses micro-electro-mechanical systems (MEMS) technology to develop a flexible five-in-one (temperature, flow, voltage, current, and pressure) microsensor, which is embedded in vanadium redox flow battery, for real-time sensing. Its advantages include: (1) Small size and the simultaneous measurement of five important physical quantities; (2) elastic measurement position and accurate embedding; and (3) high accuracy, sensitivity, and quick response time. The flexible five-in-one microsensor embedded in the vanadium redox flow battery can instantly monitor the changes in different physical quantities in the vanadium redox flow battery during charging; as such, optimum operating parameters can be found out so that performance and life can be enhancec.

## 1. Introduction

The National Aeronautics and Space Administration (NASA) proposed the redox flow battery system in 1979. This technology allows the enhancement of large-scale high performance Electrical Energy Storage (EES) [1]. Skyllas-Kazacos et al. [2] proposed the vanadium redox flow battery (VRFB) in 1986, which is extensively regarded as the most advanced and the most promising redox battery system because it overcomes the cross contamination problem resulting from the other redox flow batteries. In comparison to conventional batteries, the VRFB has such advantages as flexible design, high efficiency, high power, momentary charge, long cycle life, and high safety [3,4]. Several authors have conducted theoretical simulations and external intrusive measurements on the internal signals of vanadium flow batteries. Bhattarai [5] proposed a new method of segmenting a conventional flow cell which, for the first time, splits up both the porous felt as well as the current collector. This dual segmentation results in a higher resolution and the distinct separation of voltages between flow inlet to outlet. Gandomi [6] proposed a local potential measurement technique and applied it to all-vanadium redox flow batteries to determine the potential distribution within multilayer electrodes of the battery. There is very little information on the signal measurement inside the vanadium flow battery, and the commercially available sensor is still too large to be embedded in the vanadium flow battery; now, it is still difficult for commercially available sensors to be put in the electrochemical environment of a vanadium flow battery.

**Figure 1:**
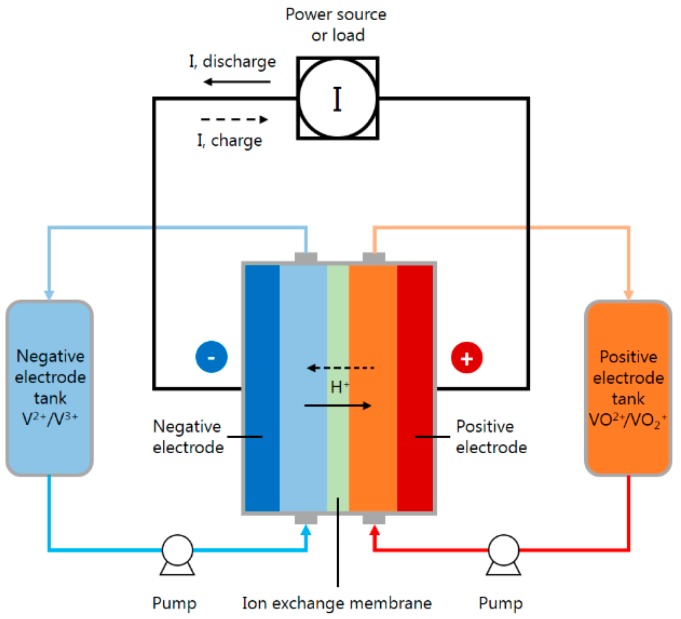
Schematic diagram of the vanadium redox flow battery (VRFB) system, adapted from [7].

The electrolyte solution of the VRFB is a sulphuric acid solution containing vanadium ions. The positive and negative electrolytes are stored in different storage tanks which are fed into the battery body by motor during battery charge/discharge. Figure 1 is the schematic diagram of the VRFB system [7]. In the actual operation process, when the battery is fully charged, the vanadium ions in the positive storage tank are oxidized and stored in the form of VO^2+^ (there is no VO^2+^); meanwhile, the V^3+^ in the negative storage tank is reduced to V^2+^. In the discharge process, the V^2+^ in the negative storage tank is oxidized to the form of V^3+^, the released electrons get to the external current collector through the collector plate, and the hydrogen ions in the battery move from the negative terminal to the positive terminal through the proton exchange membrane so as to maintain the electric neutrality of electrolyte [8]. The overall equations are expressed as follows:

Positive equation:

VO^2+^ + H_2_O ↔ VO^2+^ + 2H^+^ + e^−^ (1)

Negative equation:

V^3+^ + e^−^ ↔ V^2+^ (2)

Full equation:

V^3+^ + VO^2+^ + H_2_O ↔ V^2+^ + VO^2+^ + 2H^+^ (3)

## 2. Sensing Principle of Flexible 5-In-1 Microsensor

### 2.1. Micro Temperature Sensor

**Figure 2:**
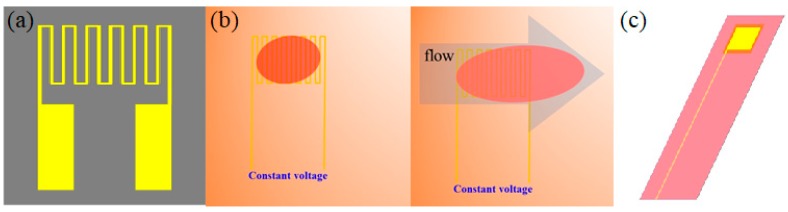
Schematic diagram of micro sensor: (**a**) Micro temperature sensor; (**b**) micro flow sensor; (**c**) micro voltage sensor.

The principle of a resistance temperature detector (RTD) is that the resistance value of metallic conductors increase with ambient temperature. The ambient temperature is calculated by extracting the resistance of structure. Figure 2a is the schematic diagram of the resistance micro temperature sensor structure used in this study. Since Charles William Siemens used it for the first time in the middle of the 19th century, the RTD has been used in most temperature measurement systems extensively.

For most of metals, if the temperature is higher than Debye temperature, the relationship between resistance *R* and temperature *T* is expressed as Equation (4).

*R* = *R*_0_ (1 + *AT* + *BT*^2^ + *C*′*T*^3^) (4)

where *R* is metallic resistance; *R*_0_ is the metallic resistance at 0 °C; *A*, *B,* and *C*′ are material constants; and *T* is the ambient temperature.

When the ambient temperature is higher than the freezing point of water, *BT*^2^ and *C*′*T*^3^ terms can be omitted, and Equation (4) can be reduced to Equation (5) according to the temperature coefficient of resistance (TCR) of material.

*R* ≈ *R*_0_ (1 + *αT*) (5)

wherein *α* is the TCR.

As Au has positive temperature coefficient (PTC), when the ambient temperature rises, the resistance value of RTD increases. This characteristic results from the TCR, defined as Equation (6).

α ≈ 1/R_0_ × dR/dT (6)

The specific relationship between resistance and temperature can be established by Equations (5) and (6) [9,10].

### 2.2. Micro Flow Sensor

The main sensing structure of hot-wire micro flow sensor is thermal resistance heater. Its major advantage is its small size—its diameter is only tens to hundreds of micrometers, which can hardly influence the measured fluid, as shown in Figure 2b.A constant voltage input generates the heat source, which leads to a stable temperature field being formed. In the flow field, the temperature field generated by the heater varies with the forced heat convection of fluid. If the external heat for the heater is constant, the resistance value of heater decreases as the fluid flow and the heat carried away increase. The heat for the hot wire is controlled, the temperature difference between hot wire and flow remains constant, the heating power increases fluid flow, and the flow can be converted into electric signal output by constant temperature circuit design. In other words, the hot-wire micro flow sensor is a microsensor with a design based on the positive correlation between the thermal energy dissipation rate of hot wire and the fluid flow.

According to King’s law, the relationship between thermal energy dissipation rate and fluid flow rate is expressed as Equation (7) [11].

*Q* = *I*^2^ × *R* = *I* × *V* = (*A* + *B* × *U^n^*) (*T*_s_ − *T*_o_) (7)

where *Q* is the electric power supplied from the external power supply; *U* is the flow rate of fluid; *n* is the coefficient of heat correlation between electric power *Q* and flow rate *U* (about 0.5 according to experiment); *T*_s_ is the hot wire temperature; and *T*_o_ is the temperature of the inlet fluid.

If a steady voltage is given in Equation (7), and the heat changes to some extent, the current changes at the same time. As such, this study extracts the current to establish the relationship between current and liquid flow instead of calculating the flow from the power and temperature by relation (7), so that any error values resulting from the equipment accuracy and calculation process can be reduced effectively [12].

### 2.3. Micro Voltage Sensor

The electrode form of the micro voltage sensor structure used in this study is a thin conductor electrode structure, as shown in Figure 2c. The principle is that two thin conductors contact both ends of analyte, and, so as to measure the voltage difference between the two thin conductors, the analyte is given a firm power. In terms of voltage measurement, the micro voltage sensor was embedded in the cathode terminal of the VRFB, with the voltage between anode terminal press plate and micro voltage sensor measured by an instrument; the difference resulted from the voltages of two press plates is measured by external instrument.

### 2.4. Micro Current Sensor

**Figure 3:**
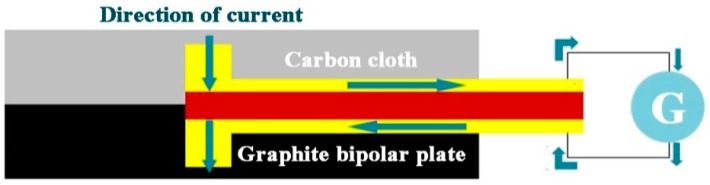
Schematic diagram of sensing principle of micro current sensor.

The micro current sensor is a miniaturized galvanometer probe of an extension conductor. Two probes face towards two sides of foil substrate. The sensing area is exposed at the foremost end, and the rest of conductor is covered with insulating layer, as shown in Figure 3. The micro current sensor penetrates into the VRFB, which is connected to the current detector to form a serial circuit in order to measure the local current in the VRFB.

### 2.5. Micro Pressure Sensor

**Figure 4:**
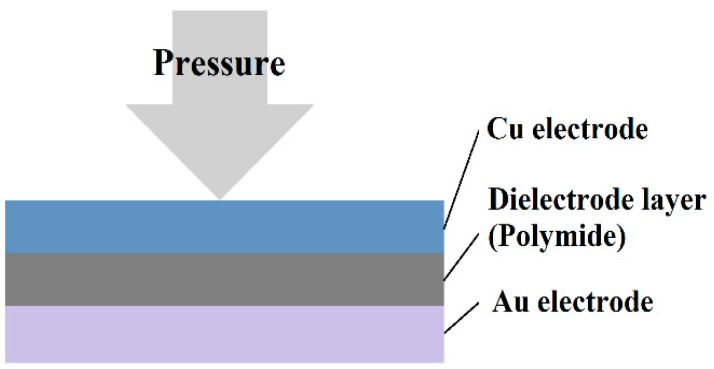
Schematic diagram of sensing principle of micro pressure sensor.

The structure of the capacitive micro pressure sensor is a nonconducting dielectric layer sandwiched in between two parallel electrodes (Figure 4). The capacitance value is calculated as Equation (8).

(8) $$\Delta {C=\varepsilon }_{r}\varepsilon_{0}\frac{A}{\Delta d}$$

where $\varepsilon_{r}$ is the dielectric constant of material, $\varepsilon_{0}$ is constant 8.854 × 10^−12^ (F/m), *A* is the projection overlapping area of two parallel electrodes, and Δ*d* is the rate of change in vertical distance between two parallel electrodes. According to Equation (8), the dielectric constant and the projected area of two parallel electrodes only influence the initial capacitance value; the rate of change in capacitance is influenced mainly by the rate of change in the distance between two parallel electrodes.

If one electrode structure is a deformable membrane, the dielectric layer is the micro pressure sensor of air or vacuum hollow structure. The capacitance is calculated as Equation (9).

(9) $$\Delta C=\iint^{\;}\frac{\varepsilon }{d-w\left(x,\;y\right)}dx\;dy-C_{0}$$

where *w*(*x*,*y*) is the function of *x*−*y* deformation of membrane.

In order to enable the capacitive micro pressure sensor to display linear response, increase the sensitivity, and consider the process convenience, a Fujifilm Durimide^®^ PI 7320 with the appropriate rigidity, dielectric constant, E-modulus, and process convenience was used as the dielectric layer of the micro pressure sensor. As the cavity between two parallel plate electrodes is replaced by polymers, the membrane electrode deforms averagely. In addition, the dielectric constant of PI 7320 is 3.2, whereas the dielectric constant of air is 1. The initial capacitance of micro pressure sensor is higher, and the rate of change in capacitance value is increased so that the sensitivity is increased—all of which is favorable for the correction and measurement of the micro pressure sensor [13].

## 3. Fabrication and Correction of Flexible 5-in-1 Microsensor

### 3.1. Design of Flexible 5-In-1 Microsensor

**Figure 5:**
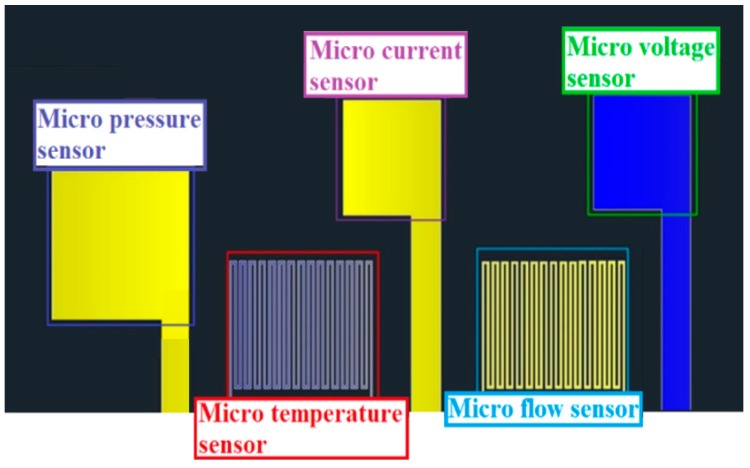
Integrated design drawing of flexible five-in-one microsensor.

Figure 5 is the integrated design drawing of the flexible five-in-one microsensor designed in this study. The temperature sensing area is 750 μm × 600 μm; the flow sensing area is 750 μm × 600 μm; the voltage sensing area is 600 μm × 600 μm; the current sensing area is 600 μm × 600 μm; and the pressure sensing area is 850 μm × 850 μm. The development was completed by using MEMS technology. Five important physical quantities can be detected simultaneously, and the optimal material and process were selected to avoid the flexible five-in-one microsensor being damaged by the harsh electrochemical environment in the VRFB.

### 3.2. Process of Flexible 5-In-1 Microsensor

**Figure 6:**
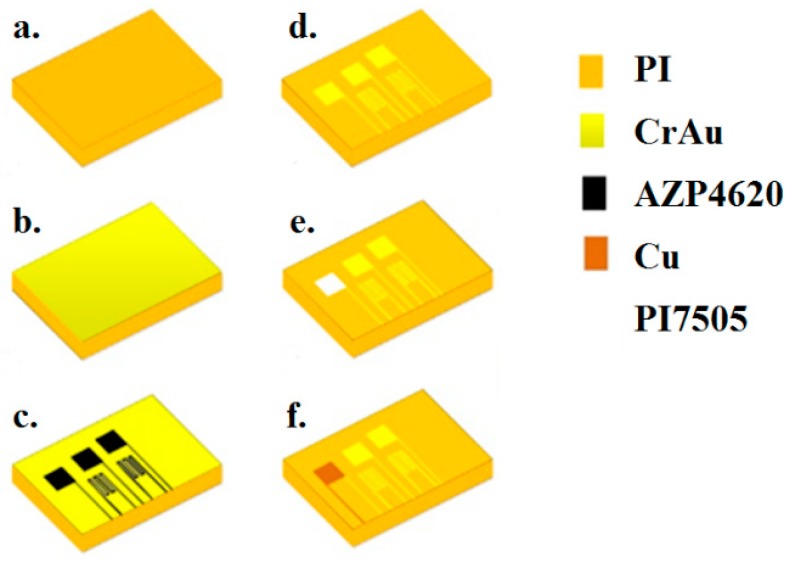
Process of flexible five-in-one microsensor.

This study used MEMS technology to integrate temperature, flow, voltage, current, and pressure sensing structures. As the electrolyte solution of the VRFB contains sulfuric acid, the flexible five-in-one microsensor must be soaked in electrolyte for a long time in operation. Therefore, this study used Polyimide (PI), a thin-film material with good acid resistance, as the flexible substrate of five-in-one microsensor, since its thickness is smaller than 50 μm. The surface micromachining technology superposes and processes multiple films to form a microstructure. This technology does not damage the substrate in comparison to bulk micro-machining, and it meets the characteristics of a structure for flexible five-in-one microsensor. As such, this study uses such surface micromachining technologies as deposition, lithography, wet etching, and metal lift off of micro-electromechanical process technology to integrate five sensing structures into the PI film. The fabrication process is shown in Figure 6.

### 3.3. Correction of Flexible 5-In-1 Microsensor

**Figure 7:**
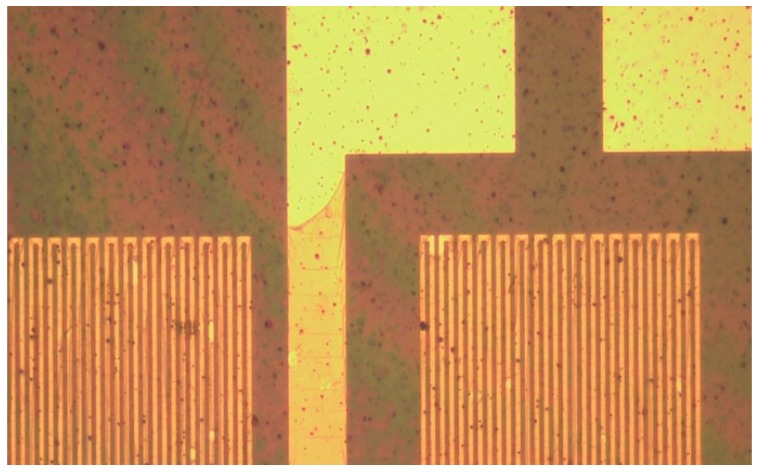
Completed flexible microsensor sensing structure.

When the sensing structure is integrated into the PI film successfully (Figure 7), the temperature and flow of the flexible five-in-one microsensor are corrected, and the reliability is validated.

**Figure 8:**
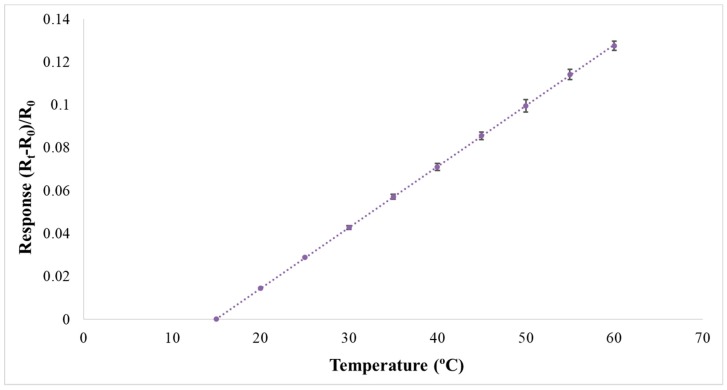
Correction curve of micro temperature sensor.

**Figure 9:**
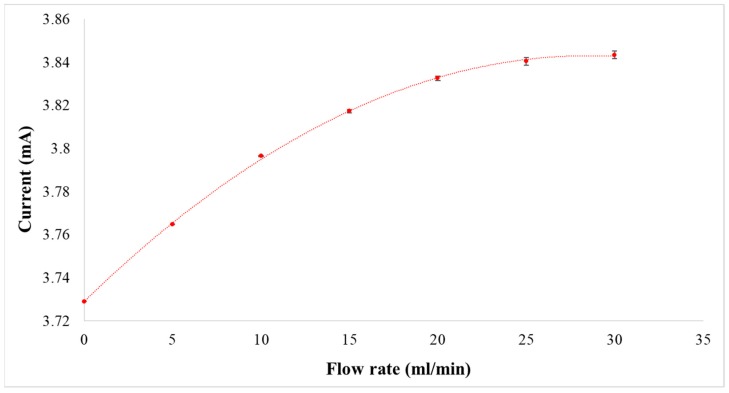
Correction curve of micro flow sensor.

The flexible five-in-one microsensor was connected to the NI PXI 2575 data capture equipment of National Instruments (NI) to extract microsensor data instantly. The resistance and current of micro temperature and flow sensors were extracted; the LabVIEW system was used to design software measurement and control system; the signals were processed, analyzed, and exported to the computer; and the temperature and flow correction curves were drawn in order to guarantee the accuracy of temperature and flow measurement. The temperature sensor was corrected by using the Hung ta HT-8045A Environmental Chamber. The correction range of temperature is 15 °C–60 °C. The micro temperature sensor has an accuracy of better than 0.5 °C. The flow sensor was corrected by using the German KNF STEPDOS 03RC. The correction range of flow rate is 0~30 mL/min. The micro flow rate sensor has an accuracy of ±2%. The results are shown in Figure 8 and Figure 9. The flexible five-in-one microsensor temperature accuracy is ≦0.5 °C; flow accuracy is ≦0.1 mL/min; voltage accuracy is ≦1 mV; current accuracy is ≦1 μA; response time is 1 ms; and temperature microsensor sensitivity is 2 × 10^−3^.

## 4. Real-Time Microscopic Diagnosis in the VRFB

### 4.1. Location of the Flexible 5-In-1 Microsensor

**Figure 10:**
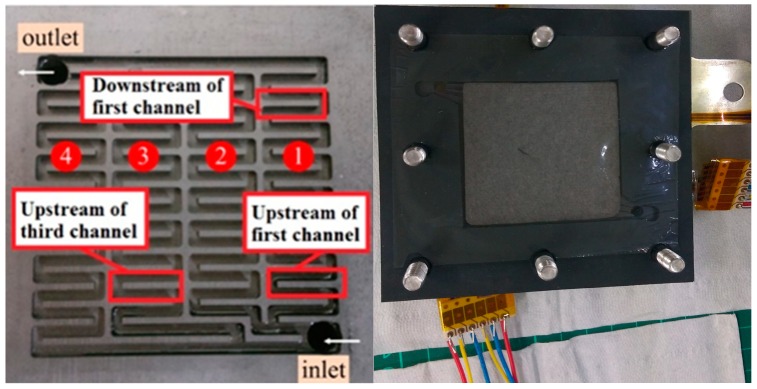
Numbers and locations of anode channels of the VRFB.

In order to completely observe the changes in internal and local physical quantities of the VRFB without influencing the performance of the VRFB, the flexible five-in-one microsensor was embedded in different runner positions under different operating conditions for comparison. Figure 10 shows the numbers of anode channels of the VRFB. The sensors are located in the first and the third channel. When the flexible five-in-one microsensor was embedded, the VRFB was closed uniformly by the closing pressure of end plate so that the signal of flexible five-in-one microsensor could be captured stably.

### 4.2. VRFB Precharge

**Figure 11:**
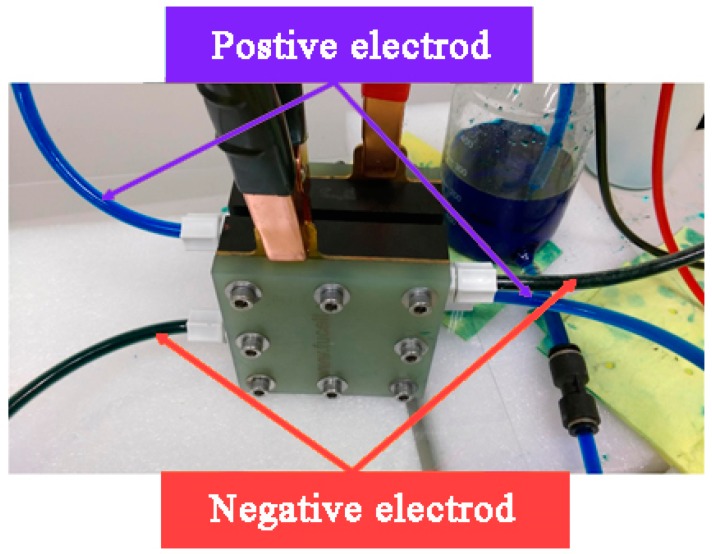
Color states of positive and negative vanadium electrolytes after precharge.

The first cause for the exchange of positive and negative ions of electrolyte is that the V^3+^ and V^4+^ exist in the solution simultaneously in the initial state of vanadium electrolyte (known as V^3.5+^, the color of which is between blue and green). The anode must be transformed to V^4+^ (azure blue), the cathode must be transformed to V^3+^ (dark green)—as shown in Figure 11—and then the VRFB can be charged/discharged normally.

**Figure 12:**
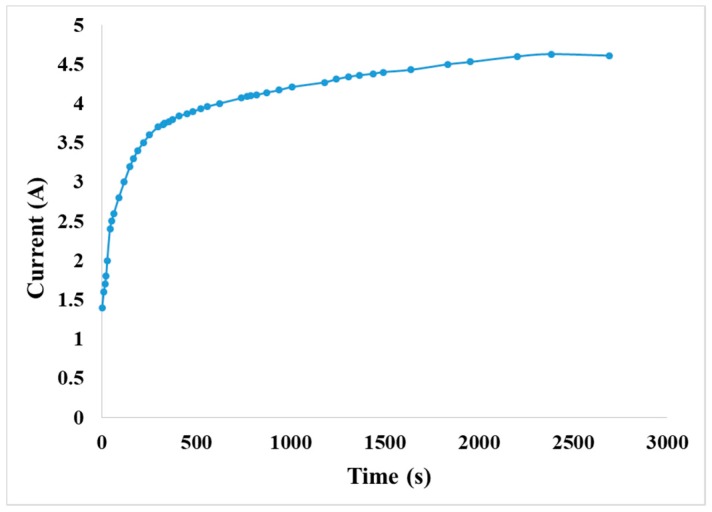
Voltage curve of the VRFB ion exchange.

The power supply was set as constant voltage of 2.5 V for precharge, the current was set as the maximum limit of power supply, and the precharge was finished when the color of vanadium electrolyte is changed apparently. Figure 12 shows the voltage curve of exchange of positive and negative ions of the VRFB.

### 4.3. VRFB Charge Test

After precharge, the anode and cathode of the VRFB were transformed to V^4+^ and V^3+^. Constant current 1 A was used for charge, the positive and negative electrolyte storage tanks were filled with 200 mL vanadium electrolyte, respectively, and the state of charge (SOC) was 100% when the voltage reaches its maximum.

**Figure 13:**
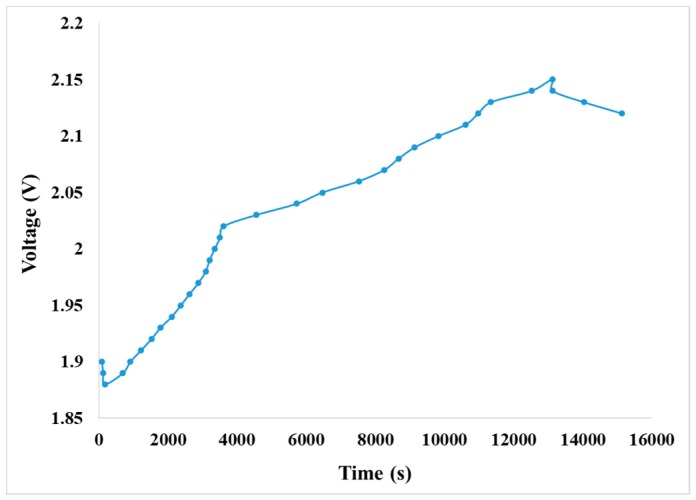
1 A constant current charging curve.

According to the curve trend, the battery charging state is regarded as 100% when the voltage reaches 2.15 V. The curve descends in the first 200 s of initial charge when the carbon felt is electrified and relatively hydrophilic. Then, more vanadium ions are contacted, so the voltage drops in the beginning. The curve rises rapidly from 200 s–4000 s, a change which may happen because lots of vanadium ions are in redox. The curve rises slowly after 4000 s; this change may happen because the vanadium electrolyte is fed circularly, most vanadium ions have completed their reaction, and a few unreacted vanadium ions perform redox reaction continuously. The charging curve of the VRFB is shown in Figure 13.

### 4.4. Real-Time Microscopic Diagnosis in the VRFB

#### 4.4.1. Observation on Internal Temperature of the VRFB

**Figure 14:**
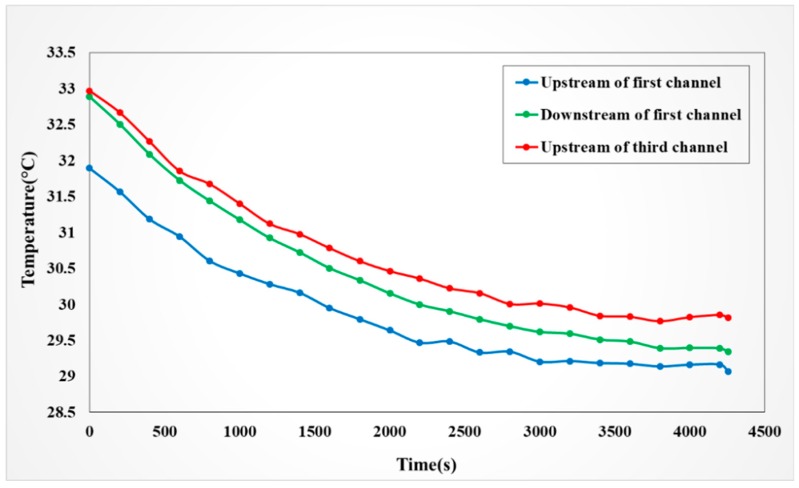
Temperature changes in battery in 1A constant current charging process.

The redox reaction is performed when the vanadium electrolyte of the VRFB is charging. The redox process generates heat, so the temperature of vanadium electrolyte rises. This study used the micro temperature sensor in the flexible five-in-one microsensor to measure the temperature change during the 1 A constant current charging of vanadium electrolyte, as shown in Figure 14. It was observed that the temperature difference between the downstream and upstream of the first channel decreased as the reaction time extends; this may be because the first channel is far from the middle reaction area but close to the bipolar plate without a channel, and the heat sinking is favorable. There are less late reactants, the generated heat is reduced a lot, and the temperature difference is reduced gradually. The temperature of the third channel was higher than the temperature in the same position of the first channel, which may be because the heat is generated continuously in the center of reaction area, the excess heat is carried away only by vanadium electrolyte, and there is no extensive heat transfer through the bipolar plate.

#### 4.4.2. Observation on Internal Flow of the VRFB

**Figure 15:**
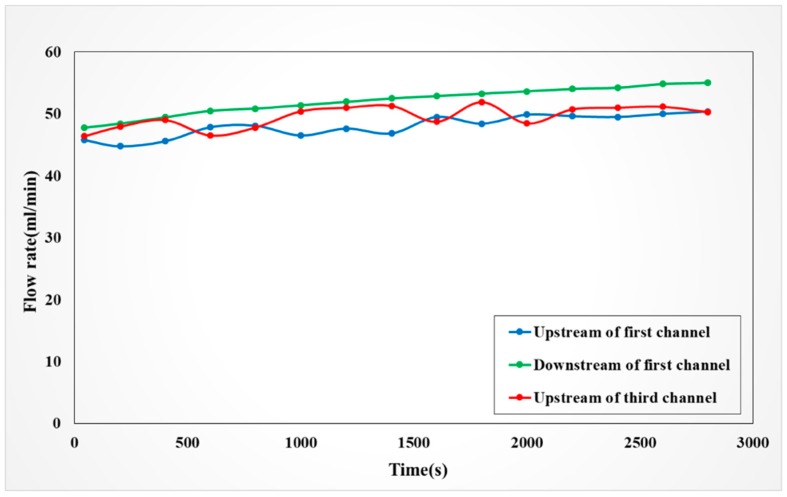
Flow in anode of battery in 1A constant current charging process.

In the SOC, the positive vanadium electrolyte increases, thus disregarding the initial split flow rate of different channels. The first channel may absorb and distribute the imported and generated vanadium electrolyte to various channels, as the vanadium electrolyte is formed and the carbon felt becomes hydrophilic. The flow in the downstream of the first channel is higher than that in the upstream. However, when the vanadium electrolyte enters the bipolar plate of the VRFB, it is well-delivered to the third channel. The formation of vanadium electrolyte may increase the fluid in the third channel, which can cause the flow rate to decrease, as shown in Figure 15.

#### 4.4.3. Observation on Internal Voltage of the VRFB

**Figure 16:**
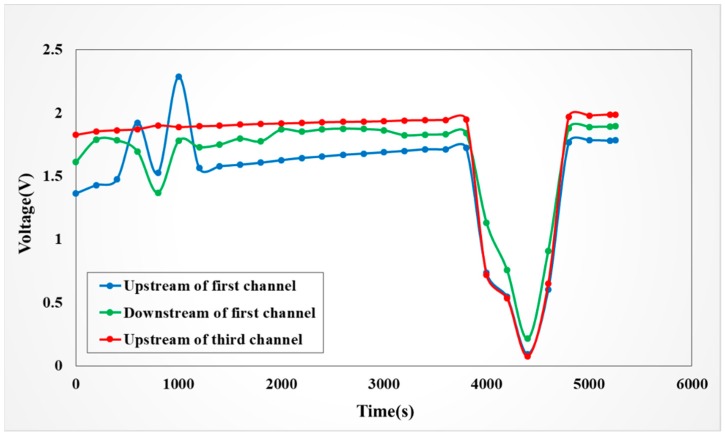
Voltage in anode of battery in 1A constant current charging process.

Figure 16 shows the internal and local voltage differences when the VRFB anode is charging. In the first channel and the third channel at the beginning and around the end, it is obvious that the voltages fluctuate greatly before and after the reaction and at the same time. It can be inferred that when the vanadium redox battery charge has both just started and almost finished, the voltage is unstable. However, the voltage is relatively stable in the other charging periods because the SOC in the battery is stable.

#### 4.4.4. Observation on Internal Current of the VRFB

**Figure 17:**
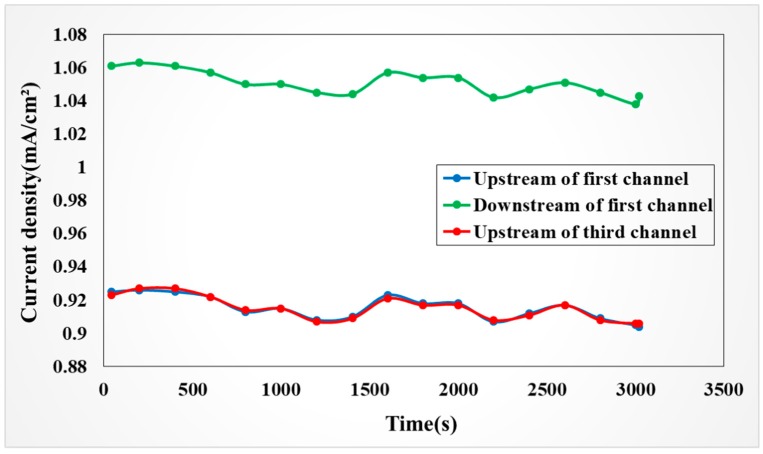
Current density in anode of battery in 40 mA/cm^2^ constant current charging process.

As shown in Figure 17, when a constant current of 40 mA/cm^2^ was used for charge, the current density in the VRFB was stable. Even if the downstream and upstream have some current density differences, the current density difference is even—there is no abrupt change.

## 5. Conclusions

This study used MEMS technology to successfully integrate micro temperature, flow, voltage, current, and pressure sensors into a 50 μm thick PI film substrate. This flexible five-in-one microsensor has five functions and such advantages as small thickness, small structural area, high sensitivity, real-time measurement, and arbitrary placement.

The flexible five-in-one microsensor can be embedded in the battery anode runner plate without influencing the sealing condition of the VRFB. The NI PXI 2575 data acquisition unit captures the information of five internal and local physical quantities of the VRFB successfully in the VRFB charging process. It is observed that one channel has a very slight deviation before and after the reaction. This deviation can be provided to fabricators to enhance the battery technology to prolong the life of the VRFB.
